# Aberrant monocytopoiesis drives granuloma development in sarcoidosis

**DOI:** 10.1093/intimm/dxad054

**Published:** 2023-12-26

**Authors:** Ryosuke Hiranuma, Ryota Sato, Kiyoshi Yamaguchi, Satoshi Nakamizo, Kenichi Asano, Takuma Shibata, Ryutaro Fukui, Yoichi Furukawa, Kenji Kabashima, Kensuke Miyake

**Affiliations:** Division of Innate Immunity, Department of Microbiology and Immunology, The Institute of Medical Science, The University of Tokyo, Tokyo, Japan; Division of Innate Immunity, Department of Microbiology and Immunology, The Institute of Medical Science, The University of Tokyo, Tokyo, Japan; Division of Clinical Genome Research, The Institute of Medical Science, The University of Tokyo, Tokyo, Japan; Department of Dermatology, Kyoto University Graduate School of Medicine, Kyoto, Japan; Alliance Laboratory for Advanced Medical Research, Kyoto University Graduate School of Medicine, Kyoto, Japan; Laboratory of Immune Regulation, School of Life Sciences, Tokyo University of Pharmacy and Life Sciences, Tokyo 192-0392, Japan; Division of Innate Immunity, Department of Microbiology and Immunology, The Institute of Medical Science, The University of Tokyo, Tokyo, Japan; Division of Innate Immunity, Department of Microbiology and Immunology, The Institute of Medical Science, The University of Tokyo, Tokyo, Japan; Division of Clinical Genome Research, The Institute of Medical Science, The University of Tokyo, Tokyo, Japan; Department of Dermatology, Kyoto University Graduate School of Medicine, Kyoto, Japan; Skin Research Institute of Singapore (SRIS) and A*STAR Skin Research Labs (A*SRL), Agency for Science, Technology, and Research (A*STAR), 8A Biomedical Grove, IMMUNOS Building, Biopolis, Singapore; Division of Innate Immunity, Department of Microbiology and Immunology, The Institute of Medical Science, The University of Tokyo, Tokyo, Japan

**Keywords:** macrophage, mTORC1

## Abstract

In sarcoidosis, granulomas develop in multiple organs including the liver and lungs. Although mechanistic target of rapamycin complex 1 (mTORC1) activation in macrophages drives granuloma development in sarcoidosis by enhancing macrophage proliferation, little is known about the macrophage subsets that proliferate and mature into granuloma macrophages. Here, we show that aberrantly increased monocytopoiesis gives rise to granulomas in a sarcoidosis model, in which Tsc2, a negative regulator of mTORC1, is conditionally deleted in CSF1R-expressing macrophages (*Tsc2*^*csf1rΔ*^ mice). In *Tsc2*^*csf1rΔ*^ mice, common myeloid progenitors (CMPs), granulocyte-monocyte progenitors (GMPs), common monocyte progenitors / monocyte progenitors (cMoPs / MPs), inducible monocyte progenitors (iMoPs), and Ly6C^int^ CX3CR1^low^ CD14^−^ immature monocytes (iMOs), but not monocyte-dendritic cell progenitors (MDPs) and common dendritic cell progenitors (CDPs), accumulated and proliferated in the spleen. Consistent with this, monocytes, neutrophils, and neutrophil-like monocytes increased in the spleens of *Tsc2*^*csf1rΔ*^ mice, whereas dendritic cells did not. The adoptive transfer of splenic iMOs into wild-type mice gave rise to granulomas in the liver and lungs. In these target organs, iMOs matured into Ly6C^hi^ classical monocytes/macrophages (cMOs). Giant macrophages (gMAs) also accumulated in the liver and lungs, which were similar to granuloma macrophages in expression of cell surface markers such as MerTK and SLAMF7. Furthermore, the gMA-specific genes were expressed in human macrophages from sarcoidosis skin lesions. These results suggest that mTORC1 drives granuloma development by promoting the proliferation of monocyte/neutrophil progenitors and iMOs predominantly in the spleen, and that proliferating iMOs mature into cMOs and then gMAs to give rise to granuloma after migration into the liver and lungs in sarcoidosis.

## Teaser

How monocytopoiesis regulates granulomas

## Introduction

Granulomas, nodules consisting of various immune cells including macrophages, B cells, and T cells, develop in innate immune responses to pathogens and foreign bodies (1). Macrophages change their shape in several ways, ranging from epithelioid and lipid-laden cells to multinuclear giant cells to form granulomas. In sarcoidosis, an immune disorder of unknown etiology, granulomas develop in multiple organs, including the lungs, heart, skin, and liver, without any apparent infection (2). In most cases, granulomas disappear spontaneously or after treatment with corticosteroids or cytotoxic agents. However, granulomas continue to develop in refractory sarcoidosis, which leads to tissue damage.

In mycobacterial infections, TLR2 ligands act on common monocyte progenitors (cMoPs) in the bone marrow (BM) to differentiate into multinucleated giant cells (3, 4), suggesting that granuloma development begins early during monocyte/macrophage development. In *Escherichia coli* infection, premonocytes proliferate in circulation and peripheral organs to replenish the peripheral monocyte/macrophage pool (5). These findings demonstrated that infections upregulate monocytopoiesis to support peripheral monocyte/macrophage responses. However, little is known regarding the role of monocytopoiesis in sarcoidosis.

Various signals emanating from TLRs and cytokine receptors drive granuloma formation (1). For example, TLR2-ligation activates DNA damage responses to initiate macrophage transformation into multinucleated giant cells (3). In addition, the mechanistic target of rapamycin complex 1 (mTORC1) is a key signaling molecule for granuloma development in sarcoidosis (6). mTORC1 senses nutrients such as amino acids to coordinate cell metabolism and proliferation (7). mTORC1 is negatively regulated by the Tuberous Sclerosis (TSC) complex, which consists of TSC1, TSC2, and TBC1 domain family member 7 (TBC1D7) (8). Loss-of-function mutations in *Tsc2* in monocytes/macrophages result in the homeostatic activation of mTORC1, leading to the formation of granulomas in the lungs and liver (6). In patients with the progressive form of sarcoidosis, TSC1 mRNA levels are significantly decreased and mTORC1 is activated (6).

Although mTORC1 activation in macrophages drives proliferation (6), the monocyte/macrophage subset that proliferates in response to Tsc2-deficiency remains unclarified.

In addition to proliferation, monocytes mature into granuloma macrophages. In mycobacterial granuloma formation, macrophage epithelial reprogramming enables epithelium-like adhesion through E-cadherin expression (9), whereas the DNA damage response promotes polypoid macrophage differentiation (3). In cardiac sarcoidosis, single-cell RNA-seq analyses have demonstrated several types of macrophages in granulomas, such as resident macrophages, monocytes, and glycoprotein nonmetastatic melanoma protein B (GPNMB)^+^ giant cells (10). GPNMB is a marker for multinucleated giant cells (11). Granuloma macrophages are characterized by these changes in gene expression.

In this study, we analyzed mutant mice in which *Tsc2* was deleted under the *Csf1r*-Cre driver to investigate the cellular and molecular mechanisms underlying granuloma development. Tsc2-deficiency in CSF1R-expressing myeloid cells resulted in increased proliferation and accumulation of monocyte progenitors (MPs) such as common myeloid progenitors (CMPs), granulocyte-monocyte progenitors (GMPs), common monocyte progenitors / monocyte progenitors (cMoPs / MPs), inducible monocyte progenitors (iMoPs), and of Ly6C^int^ CX3CR1^low^ CD14^−^ immature monocytes (iMOs) in the spleen. The accumulated iMOs migrated to the liver and lungs, giving rise to granulomas. Giant macrophages (gMAs), which were similar to granuloma macrophages, accumulated in the liver, lung, and BM. Human macrophages, similar to gMAs in terms of gene expression, were found in sarcoidosis skin granulomas. These results suggest that aberrantly enhanced monocytopoiesis drives the development of granuloma in sarcoidosis.

## Methods

### Mice

C57BL/6 *Tsc2*^*flox/flox*^ and *Csf1r-Cre* mice were obtained from the Jackson Laboratory. All animals were housed in SPF facilities at the Institute of Medical Science, University of Tokyo (IMSUT). The approvals to perform experiments using recombinant DNA and animals were obtained by the Institutional committees (#PA17-84, #PA22-43, #K19-25, #K19-26).

### Cell culture

BM-derived macrophages were isolated from BM cells. The cells were cultured in RPMI containing 10% fetal calf serum (FCS), 2 mM l-Glutamine (Gibco), 50 µM 2-ME (Nacalai Tesque, Osaka, Japan), and 0.1 µg/mL M-CSF (PeproTech, Inc.).

### Reagents

The 5-ethynyl-2ʹ-deoxyuridine (EdU) used in the *in vitro* and *in vivo* proliferation assays was purchased from Tokyo Chemical Industry Co. (Tokyo, Japan). RPMI1640 was purchased from Nacalai Tesque. Lipid A purified from *Salmonella minnesota* (Re-595) was purchased from Sigma–Aldrich (Merck, Darmstadt, Germany). Brefeldin A was purchased from BioLegend (San Diego, CA, USA). pHrodo Green *E. coli* bioparticles Conjugate for Phagocytosis was purchased from Invitrogen.

### Antibodies

Monoclonal anti-mouse CD11b (clone M1/70), CX3CR1 (clone SA011F11), F4/80 (clone BM8), NK1.1 (clone PK136), Siglec-F (clone S17007L), FcγRIV (clone 9E9), Ly6C (clone HK1.4), CD3 (clone 17A2), CD19 (clone 6D5), CD11c (clone N418), CD45.2 (clone 104), Tim4 (clone F31-5G3), CD34 (clone HM34), CD16/32 (clone 93), CD135 (clone A2/F10), Sca-1 (clone D7), CD319 (clone 4G2), CD200R (clone OX-110), CD200 (clone OX-90), CD49e (clone 5H10-27), CD51 (clone RMV-7), CD22 (clone OX-97), Mac-2 (clone M3/38), CD14 (clone Sa14-2), and IL-6 (clone MP5-20F3) antibodies were purchased from BioLegend.

Monoclonal antibodies (mAbs) to mouse Ly6G (clone 1A8), B220 (clone RA3-6B2), CD115 (clone T38-320), CD117 (clone2B8), TER-119 (clone TER-119), and Grb2 (clone 81/GRB2) were purchased from BD biosciences (Franklin Lakes, NJ, USA). The mAbs anti-mouse MerTK (clone DS5MMER) and Tsc2 (clone SC05-59) were purchased from Invitrogen. The monoclonal anti-Tsc2 (clone D93F12) antibody was purchased from Cell Signaling Technology. The LEGENDScreen Mouse PE Kit was purchased from BioLegend.

### Histological analysis

Mouse tissues were fixed in 20% formalin neutral buffer solution. The fixed lungs and livers were embedded in paraffin wax for sectioning. The sections were subjected to hematoxylin and eosin (H&E) staining or immunohistochemistry (IHC) staining for F4/80. For IHC staining of MerTK and Slamf7, tissues were embedded in Tissue-Tek embedding medium to prepare frozen tissue blocks (Sakura Fine Technical Co., Ltd., Tokyo, Japan). Sections were visualized using an EVOS microscope (Thermo Fisher Scientific).

### Cell preparation

Blood cells were obtained from the mice using a microtube containing EDTA (Erma Inc., Tokyo, Japan). The spleens were minced onto glass slides. BM cells were collected by flushing the femurs and tibias with a 25-gauge needle. The livers and lungs were minced and processed using a gentle MACS Octo Dissociator with Heaters (Miltenyi Biotec, Bergisch Gladbach, Germany). Supernatants were filtered using a MACS SmartStrainer (pore size: 100 µM; Miltenyi Biotec) and centrifuged at 300×g for 10 min. The pellet was resuspended in Debris Removal Solution (Miltenyi Biotec) and centrifuged at 3000×g for 10 min. The cell pellet was resuspended in red blood cell (RBC) lysis buffer (BioLegend).

### Immunoblot analysis

The cells were lysed in Sample Buffer (50 mM Tris-HCl 10% glycerol, 1% SDS, and 10% 2-ME). The lysates were heated at 95°C for 5 min. The samples were separated using SDS-PAGE and subjected to immunoblot analysis. The antibodies used for immunoblot analysis were dissolved in CanGetSignals Solution 1 (Toyobo). The secondary antibodies used were proA-HRP (Cytiva) and goat anti-mouse IgG-HRP (Santa Cruz Biotechnology) dissolved in CanGetSignals Solution 2.

### Flow cytometry

Cell surface staining for flow cytometric analysis was performed using fluorescence-activated cell sorting (FACS) staining buffer (1 × phosphate-buffered saline [PBS] with 2.5% FCS and 0.1% NaN_3_). The prepared cell samples were incubated for 10 min with an unconjugated anti-mouse CD16/32 blocking mAb (clone 95) to prevent nonspecific staining in the staining buffer. The cell samples were stained with fluorescein-conjugated mAbs for 15 min on ice. For intracellular staining of Tsc2, surface-stained cells were fixed, and permeabilized using True-Nuclear Transcription Factor Buffer Set (BioLegend) then incubated with anti-Tsc2 antibody (Invitrogen) for 30 min at 4°C followed by incubation with PE-conjugated anti-rabbit IgG (BioLegend) for 30 min at 4°C. The stained cells were analyzed using an ID7000 Spectral Cell Analyzer (Sony Biotechnology, San Jose, CA, USA). All data were analyzed using the FlowJo software (BD Biosciences).

### Proliferation assay with EdU labeling

*In vitro* and *in vivo* proliferation assays were conducted using the Click-iT Plus EdU Alexa Fluor 488 Flow Cytometry Assay Kit (Invitrogen), according to the manufacturer’s instructions. The BM, spleen, liver, and lung cells were labeled by incubation in 10 µM EdU dissolved in RPMI medium for 1 h at 37°C, or by intravenous injection of 3 mg EdU dissolved in 1 × PBS 3 h before analysis. After blocking with anti-CD16/32 (clone 95) mAb, the samples were stained with fluorescent dye-conjugated mAbs. The stained samples were subsequently fixed with BD Cytofix (BD Biosciences) and permeabilized using 1 × Click-iT saponin-based permeabilization and washing reagent. Finally, EdU incorporated into the genomic DNA was stained using the Click-iT EdU reaction cocktail, followed by staining with Fxcycle Violet Stain (Invitrogen). EdU-positive cells were detected using a spectral flow cytometer ID7000 (Sony Biotechnology).

### In vitro phagocytosis assay

Spleen cells were incubated with 50 µg/mL pHrodo Green *E. coli* (Invitrogen) dissolved in RPMI medium for 1 h at 37°C. After blocking with anti-CD16/32 (clone 95) mAb, the samples were stained by fluorescent dye-conjugated mAbs. pHrodo Green positive cells were detected using a spectral flow cytometer ID7000 (Sony Biotechnology).

### In vitro cytokine production

Spleen cells were incubated with 5 µg/mL Brefeldin A and 1 µg/mL Lipid A dissolved in RPMI medium for 3 h at 37°C. After blocking with anti-CD16/32 (clone 95) mAb, the samples were stained by fluorescent dye-conjugated mAbs. Surface-stained cells were fixed and permeabilized using BD Cytofix/Cytoperm Fixation/Permeabilization Kit (BD Biosciences) then incubated with PE-conjugated anti-IL-6 antibody (BioLegend) for 1 h at 4°C. The stained cells were analyzed using a spectral flow cytometer ID7000 (Sony Biotechnology).

### Cell sorting

Cell sorting was performed using a FACS ARIA III Cell Sorter (BD Biosciences). To purify splenic iMOs and cMOs or liver mMAs and gMAs, spleen and liver cells from *Tsc2*^*Csf1rΔ*^ mice were incubated with biotinylated anti-mouse CD3 (clone 17A2) / CD19 (clone6D5)/NK1.1 (clone PK136) / Ly6G (clone aA8) / TER-119 / erythroid cells (clone Ter-119), followed by incubation with Streptavidin MicroBeads (Miltenyi Biotec). The magnetically labeled cells were removed using autoMACS (Miltenyi Biotec), and the enriched cells were stained with anti-mouse CD45.2, F4/80, CD11b, Ly6C, CD16.2, Cx3cr1, NK1.1, Ly6G, Siglec-F, and Slamf7 mAbs. CD11b^+^ NK1.1^−^ Ly6G^−^ SiglecF^−^ Ly6C^hi^ Cx3cr1^lo^ iMOs, CD11b^+^ NK1.1^−^ Ly6G^−^ SiglecF^−^ Ly6C^hi^ Cx3cr1^hi^ cMOs, CD11b^+^ NK1.1^−^ Ly6G^−^ SiglecF^−^ Ly6C^lo^ FcγRIV^hi^, Slamf7^lo^ mMAs, and CD11b^+^ NK1.1^−^ Ly6G^−^ SiglecF^−^ Ly6C^lo^ FcγRIV^hi^ SLAMF7^hi^ gMAs were sorted.

### BM transfer

C57BL/6 WT mice at 6–7 weeks of age were lethally irradiated at 4.75 × 2 Gy using MBR-1520R-4 (Hitachi Power Solutions, Ibaraki, Japan), and received 1 × 10^6^ BM cells from *Tsc2*^*fl/fl*^ or *Tsc2*^*Csf1rΔ*^ mice through intravenous routes.

### Adoptive transfer

5 × 10^5^ sorted splenic iMOs from Tsc2^Csf1rΔ^ mice were combined with 5 × 10^5^ BM cells from *Tsc2*^*fl/fl*^ mice and the cells were intravenously transferred into 6–7 week-old C57BL/6 WT mice lethally irradiated with 4.75 × 2 Gy.

### RNAseq analysis

Total RNA was extracted from sorted cells using RNeasy Mini Kits (Qiagen, Hilden, Germany) and RNA quality was evaluated using an Agilent Bioanalyzer (Agilent Technologies, Santa Clara, CA, USA). Samples with an RNA integrity number value >7.0 were subjected to library preparation. RNA-seq libraries were prepared with 1 ng of total RNA using the Ion AmpliSeq Transcriptome Mouse Gene Expression kit (Thermo Fisher Scientific), according to the manufacturer’s instructions. The libraries were sequenced with 100-bp single-end reads to a depth of at least 10 million reads per sample on the Ion Proton platform using an Ion PI Hi-Q Sequencing 200 kit and Ion PI Chip v3 (Thermo Fisher Scientific). FASTQ files were generated using the AmpliSeqRNA plug-in v5.2.0.3 in the Torrent Suite software (v5.2.2; Thermo Fisher Scientific) and analyzed using TCC-GUI software. Individual sample reads were normalized to relative log expression using the DESeq2 R library. DESeq2 was used to determine the fold changes and *P* values. Genes showing a >1.5-fold change in expression (*P* < .05) were considered significantly altered. Hierarchical clustering and heatmap were created using the TCC-GUI software. To interpret the gene expression profiles, gene set enrichment analysis (GSEA) was performed using GSEA 4.1.0, with the MSigDB KEGG gene sets. Enriched pathways were determined using FDR-adjusted *P* values of <.1. To identify the activation transcription factors, over representation analysis was conducted using Enrich R (https://maayanlab.cloud/Enrichr/) with ARCHS4 TFs Coexp.

### Single-cell RNA-seq

R (v4.1.0) and the Seurat R package (version 4.0.6) were utilized to analyze myeloid clusters from sarcoidosis and healthy skin scRNA-seq data (GSE234901). Because the data were made public, approval of the IRB at the Institute of Medical Science was not required. Both the mean gene expression and individual gene expression levels of genes upregulated in gMAs of *Tsc2 csf1rΔ* mice were analyzed in myeloid cells from human sarcoidosis granulomas.

### Statistical analysis

 A two-tailed, unpaired *t*-test with Holm-Sidak correction was used to determine significant differences between the two groups. All data are represented as mean ± SEM, and graphs were generated using PRISM.

## Results

### TSC2-deficient BM cells give rise to granuloma

**Figure 1.**
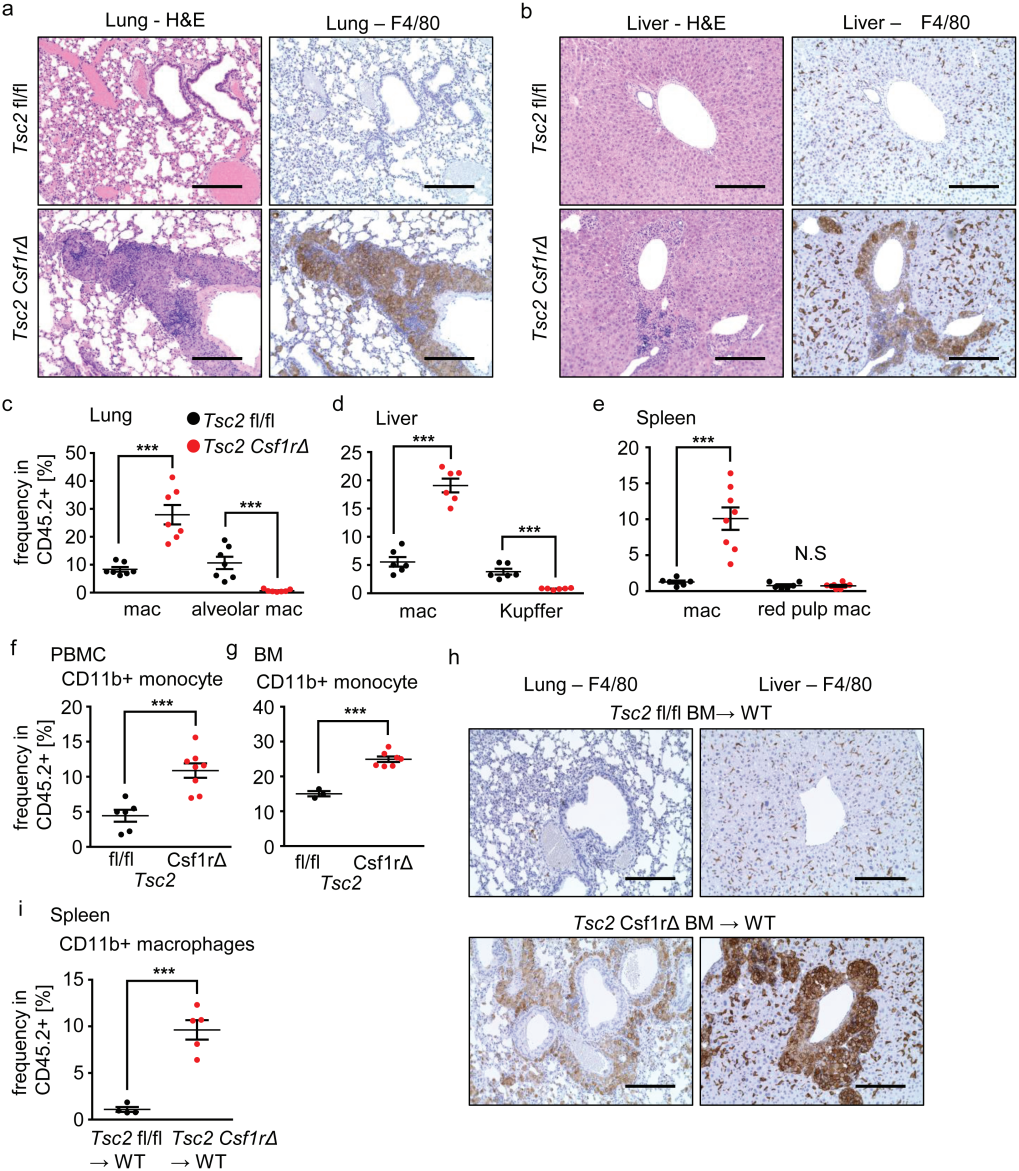
BM cells give rise to granulomas in *Tsc2*^*Csf1rΔ*^ mice. (a, b) H&E staining and F4/80 immunostaining of lung (a) and liver (b) sections of *Tsc2*^*fl/fl*^ and *Tsc2*^*Csf1rΔ*^ mice at the age of 2 months. (c–g) Percentages of CD11b^+^ Ly6G^−^ NK1.1^−^ Siglec-F^−^ monocytes/macrophages, F4/80^+^ SiglecF^+^ alveolar macrophages, and F4/80^+^ Tim4^+^ Kupffer cells in CD45^+^ hematopoietic cells in the lung, liver, spleen, peripheral blood, and BM of the indicated mice (*n* = 5–8). (h) Immunostaining for F4/80 in the lung and liver sections of the indicated BM chimeric mice. (i) Percentages of CD11b^+^ macrophages in CD45.2^+^ hematopoietic cells in the spleen of the indicated BM chimeric mice (*n* = 4–5). ***P < .001. Scale bars, 200 µm.

To delete *Tsc2* specifically in macrophages, *Tsc2*^fl/fl^ mice were crossed with *Csf1r*-Cre mice (12, 13). *Tsc2*^fl/fl^*Csf1r*-Cre mice are hereafter referred to as *Tsc2*^*csf1rΔ*^ mice, in which TSC2 protein deletion was confirmed in BM-derived macrophages (Supplementary Fig. S1a). Histological examination showed that granulomas containing F4/80^+^ macrophages were observed in the lungs and liver at 8 weeks of age in *Tsc2*^*csf1rΔ*^ mice (Fig. 1a and b), much earlier than in *Tsc2*^fl/fl^*Lyz2*-Cre mice (6). CD11b^+^ monocytes/macrophages increased in percentage in the lungs, liver, spleen, peripheral blood, and BM (Fig. 1c–g, Supplementary Fig. S1b–f), whereas tissue-resident macrophages, such as alveolar macrophages, Kupffer cells, and red pulp macrophages decreased or did not increase (Fig. 1c–e, Supplementary Fig. S1b–d). A previous report showed that BM cells give rise to granulomas in *Tsc2*^fl/fl^*Lyz2*-Cre mice (6). We also transferred BM cells from *Tsc2*^*csf1rΔ*^ mice into irradiated wild-type mice. The transfer of BM cells from *Tsc2*^*csf1rΔ*^ mice resulted in the accumulation of CD11b^+^ macrophages in the spleen and granuloma development in the lungs and liver (Fig. 1h and i). These results confirmed the previous report that TSC2-deficient BM cells give rise to granulomas (6).

### Ly6C^int^ CX3CR1^low^ immature monocytes give rise to granulomas

**Figure 2.**
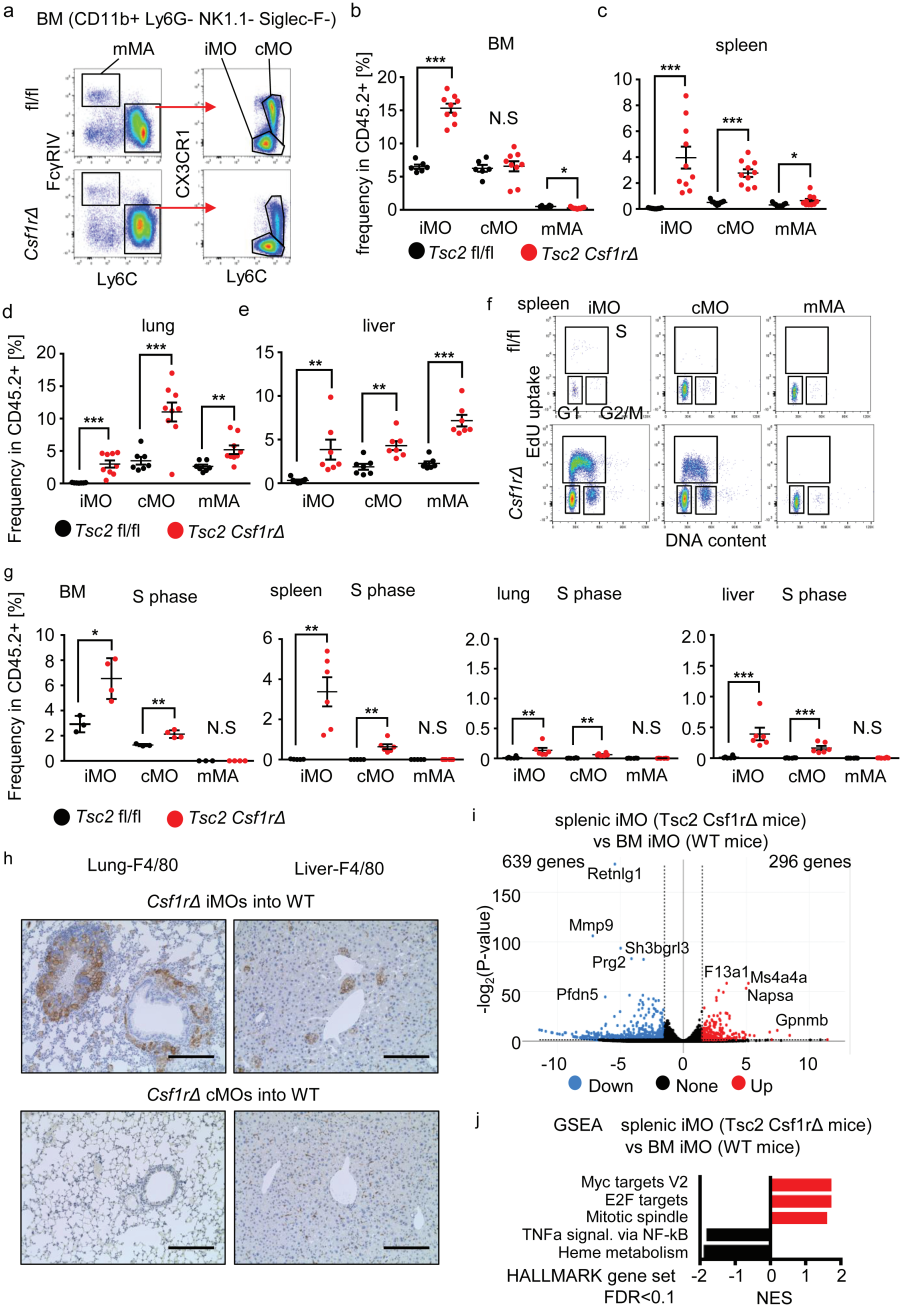
Ly6C^int^ CX3CR1^low^ iMOs give rise to granuloma. (a) Dot plots showing the expression of Ly6C, FcγRIV, and CX3CR1 in CD11b^+^ Ly6G^−^ NK1.1^−^ monocytes/macrophages in the BM of the indicated mice. (b–e) Percentages of iMOs, cMOs, and mMAs in CD45^+^ hematopoietic cells in the BM, spleen, lung, and liver of the indicated mice (*n* = 6–9). (f) Dot plots showing EdU uptake and DNA content in the splenic monocyte subsets. (g) Dots show the percentage of monocytes in the S-phase in the indicated organs (*n* = 3–6). (h) Immunostaining of F4/80 in lung and liver sections from mice that received splenic iMOs from *Tsc2*^*Csf1rΔ*^ mice with wild-type BM cells. Scale bars, 200 µm. (i) The volcano plot shows the genes expressed in splenic iMOs from *Tsc2*^*csf1rΔ*^ mice 2^1.5^ fold higher and lower than those in BM iMOs from wild-type mice. (j) GSEA of the comparison between splenic iMOs from *Tsc2*^*Csf1rΔ*^ mice and BM iMOs from wild-type mice. *P < .05, **P < .01, ***P < .001.

Next, we studied monocyte/macrophage subsets in *Tsc2*^*csf1rΔ*^ mice. Compared to *Tsc2*^*fl/fl*^ control mice, Ly6C^int^ CX3CR1^low^ iMOs were increased in the BM, spleen, lungs, and liver of *Tsc2*^*csf1rΔ*^ mice (Fig. 2a–e). Ly6C^hi^ CX3CR1^int-hi^ classical monocytes/macrophages (cMOs) increased in the spleen, lungs, and liver but not in the BM. Ly6C^low^ monocyte-derived macrophages (mMAs) increased in percentage, predominantly in the lungs and liver. These changes may result from alterations in monocyte/macrophage proliferation. Therefore, monocytes/macrophages were labeled with the thymidine analog EdU and its uptake into nuclear DNA was assessed by FACS analysis to detect monocytes/macrophages in the S phase (Fig. 2f). In *Tsc2*^fl/fl^ control mice, iMOs and cMOs in the S-phase were detected only in the BM (Fig. 2g). In *Tsc2*^csf1rΔ^ mice, the percentage of iMOs and cMOs in the S-phase in the BM was higher than that in control mice. Furthermore, iMOs and cMOs in the S-phase were detected in peripheral organs such as the spleen, lung, and liver (Fig. 2g). In contrast, we could not detect proliferation of mMAs in any organ. These results suggest that Tsc2-deficiency promoted the proliferation of iMOs and cMOs not only in the BM but also in peripheral organs such as the spleen. We next investigated whether splenic iMOs could give rise to granulomas. Splenic iMOs were sorted and adoptively transferred into irradiated wild-type mice together with wild-type BM cells. Granulomas developed in the lungs and liver of wild-type mice that received iMOs but not cMOs (Fig. 2h), suggesting that transferred iMOs migrated into the lungs and liver to form granulomas. We compared splenic iMOs *Tsc2*^*csf1rΔ*^ mice with BM iMOs from wild-type mice because splenic iMOs were too few to analyze. Transcriptome analyses revealed that splenic iMOs from expressed 296 and 639 genes by 2^1.5^ fold higher and lower, respectively, than BM iMOs from wild-type mice (Fig. 2i). GSEA demonstrated that proliferation-associated gene sets, such as Myc targets, E2F targets, and Mitotic spindle, were positively enriched in differentially expressed genes (DEGs) (Fig. 2j). These results suggest that proliferating iMOs give rise to granuloma in *Tsc2*^*csf1rΔ*^ mice.

We also studied monocyte/macrophage responses, such as phagocytic activity and IL-6 production upon lipid A stimulation. FACS analysis after treatment with pHrodo Green-conjugated *E. coli* showed that bacterial phagocytosis was impaired in iMOs, cMOs, and mMAs (Supplementary Fig. S2a). IL-6 production upon lipid A stimulation was detected only in mMAs of wild-type mice, whereas both cMOs and mMAs produced IL-6 in *Tsc2*^*csf1rΔ*^ mice (Supplementary Fig. S2b). These results demonstrate that Tsc2-deficiency impaired phagocytosis and enabled IL-6 production by cMOs.

### iMOs mature into cMOs in the liver and lungs

**Figure 3.**
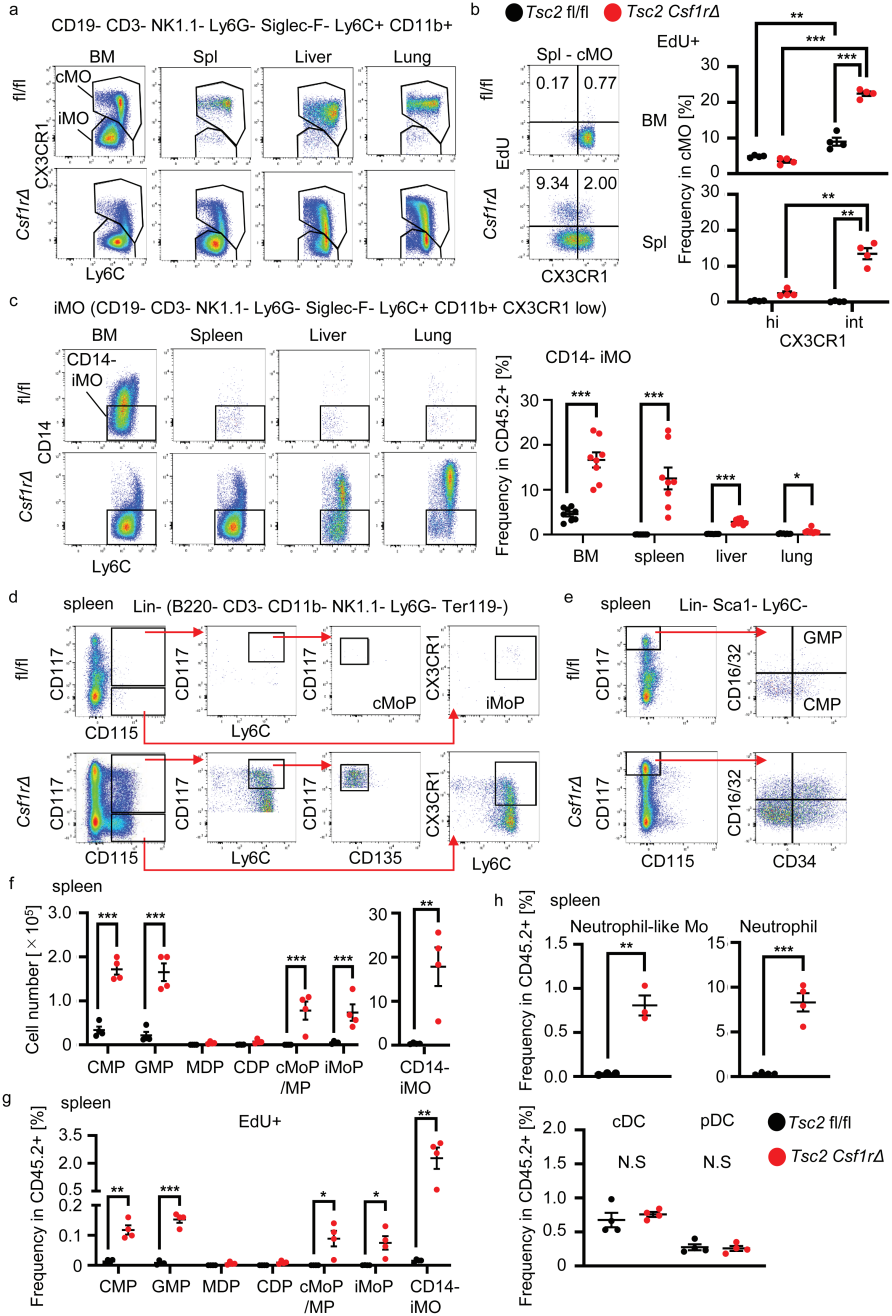
iMOs mature into cMOs after migration into the lung and liver. (a) Dot plots showing the expression of CX3CR1 and Ly6C in CD11b^+^ monocytes/macrophages in the indicated organs from the indicated mice. (b) Dot plots showing the expression of CX3CR1 and EdU uptake by splenic cMOs in the indicated mice. Percentages of EdU^+^ CX3CR1^hi^ and EdU^+^ CX3CR1^int^ cells in the BM and spleen are also shown (*n* = 4). (c) Dot plots showing the expression of CD14 and Ly6C on CD11b^+^ monocytes/macrophages in the indicated organs from the indicated mice. Percentages of CD14^−^ iMOs are also shown (*n* = 8). (d) Dot plots showing the expression of CD117, CD115, Ly6C, and CD135 in lineage-negative cells in the spleen of the indicated mice. (e) Dot plots show the expression of CD117, CD115, CD34, and CD16/32 in lineage-negative, Sca-1^−^, and Ly6C^−^ cells in the spleen of the indicated mice. (f) Total numbers of indicated monocyte progenitors and monocytes in the spleen of the indicated mice (*n* = 4). (g) Percentages of EdU^+^ monocyte progenitors/monocytes in the spleen from the indicated mice (*n* = 4). (h) Percentages of neutrophil-like monocytes, neutrophils, cDCs, and pDCs in the spleen of the indicated mice (*n* = 4). *P < .05, **P < .01, ***P < .001.

To study iMO maturation during and after migration into the lung and liver, CD11b^+^ monocytes/macrophages in the BM, spleen, lung, and liver were compared using FACS analysis. Ly6C^int^ CX3CR1^low^ iMOs and Ly6C^hi^ CX3CR1^hi^ cMOs were identified in the BM of wild-type mice (Fig. 3a). cMOs were also observed in the spleen, liver, and lungs. Compared with wild-type mice, Ly6C^hi^ CX3CR1^int^ transitional cMOs were increased in the BM and spleen of *Tsc2*^*csf1r*Δ^ mice. EdU uptake by transitional cMOs, not mature cMOs showed that transitional cMOs mainly proliferated in the spleen of *Tsc2*^*csf1rΔ*^ mice (Fig. 3b). In the liver and lungs of *Tsc2*^csf1rΔ^ mice, the percentage of iMOs decreased, whereas that of transitional cMOs increased (Fig. 3a), suggesting that iMOs migrated into the liver and lungs while or before maturing into cMOs. To further study iMO maturation into cMOs, we examined another monocyte maturation marker, CD14, whose expression increases during monocyte maturation into cMOs (5). We found that ~50% of iMOs in the BM of wild-type mice expressed CD14 (Fig. 3c). In contrast, a majority of iMOs in the BM and spleen of *Tsc2*^*csf1rΔ*^ mice did not express CD14. CD14 expression on iMOs increased in the liver and lung. These results suggest that CD14^−^ iMOs in the spleen and BM migrate into the liver and lungs to mature into CD14^+^ iMOs, and then into cMOs.

### Monocyte progenitors and iMOs proliferate and accumulate in the spleen

We found that the expression of the maturation marker CD14 in iMOs decreased in the BM of *Tsc2*^*csf1rΔ*^ mice (Fig. 3c), suggesting that BM iMOs in *Tsc2*^*csf1rΔ*^ mice were more immature than those in wild-type mice. The accumulation of CD14^−^ iMOs prompted us to study MPs. Among the BM cell populations from which erythroblasts, B cells, T cells, NK cells, monocytes, and granulocytes were excluded, we detected CD11b^−^ CD117^hi^ CD115^−^ CD34^+^ CD16/32^int^ Ly6C^−^ CMPs, CD11b^−^ CD117^hi^ CD115^−^ CD34^+^ CD16/32^+^ Ly6C^−^ GMPs, CD11b^−^ CD117^hi^ CD115^+^ CD135^+^ Ly6C^−^ monocyte-dendritic cell progenitors (MDPs), CD11b^−^ CD117^low^ CD115^+^ CD135^+^ Ly6C^−^ common dendritic cell progenitors (CDPs), CD11b^−^ CD117^hi^ CD115^+^ CD135^−^ Ly6C^+^ cMoPs/MPs, and CD11b^−^ CD117^−^ CD115^+^ Ly6C^+^ CX3CR1^+^ iMoPs (4, 14, 15) (Supplementary Fig. S3a and b). Consistent with the previous report showing CSF1R expression in CMPs and GMPs (16), FACS analysis showed reduced Tsc2 expression in MPs, except for CDPs (Supplementary Fig. S3c). Tsc2 deletion in MDPs, which is upstream of CDPs, strongly suggests Tsc2 deletion in CDPs. In the BM of *Tsc2*^*csf1rΔ*^ mice, the percentage of these progenitors was not altered, except for iMoPs, whose percentage was decreased (Supplementary Fig. S3d). Proliferation of progenitors was examined by FACS analysis using EdU staining. Compared to wild-type mice, the percentages of EdU^+^ cMoPs/MPs and EdU^+^ CD14^−^ iMOs increased whereas that of EdU^+^ iMoPs decreased (Supplementary Fig. S3e). The majority of EdU^+^ myeloid cells in the BM of *Tsc2*^*csf1rΔ*^ mice were CD14^−^ iMOs, which increased by ~2-folds in percentage compared to wild-type mice. More drastic changes were observed in the spleen of *Tsc2*^*csf1rΔ*^ mice; CMPs, GMPs, cMoPs/MPs, and iMoPs were detected in the spleen of *Tsc2*^*csf1rΔ*^ mice, but not of wild-type mice (Fig. 3d–f, Supplementary Fig. S3f). Interestingly, CDPs and MDPs were not detected in the spleen. We also observed increases in the percentages of EdU^+^ monocytes/MPs such as CD14^−^ iMOs, CMPs, GMPs, cMoPs/MPs, and iMoPs (Fig. 3g). GMPs give rise to monocytes, neutrophil-like monocytes, and neutrophils, whereas MDPs differentiate into monocytes and dendritic cells (DCs) (15, 17, 18). Consistent with the increase in GMPs but not MDPs, GMP descendants such as neutrophil-like monocytes and neutrophils increased, whereas DCs did not increase in the spleen of *Tsc2*^*csf1rΔ*^ mice (Fig. 3h, Supplementary Fig. 3g). These results suggest that TSC2-deficiency promoted the proliferation of monocyte/neutrophil progenitors as well as CD14^−^ iMOs, but not DC progenitors, in the spleen of *Tsc2*^*csf1rΔ*^ mice. MPs and cMoPs are derived from GMPs and MDPs, respectively, (Supplementary Fig. S3h) (19), and share cell surface markers with each other. Increases in neutrophil-like monocytes suggest increases in MPs in *Tsc2*^csf1rΔ^ mice (Supplementary Fig. S3h). Because we could not discriminate MPs from cMoPs, we referred to CD11b^−^ CD117^hi^ CD115^+^ CD135^−^ MPs as cMoPs/MPs.

### gMAs increased in the liver of Tsc2^csf1rΔ^ mice

**Figure 4.**
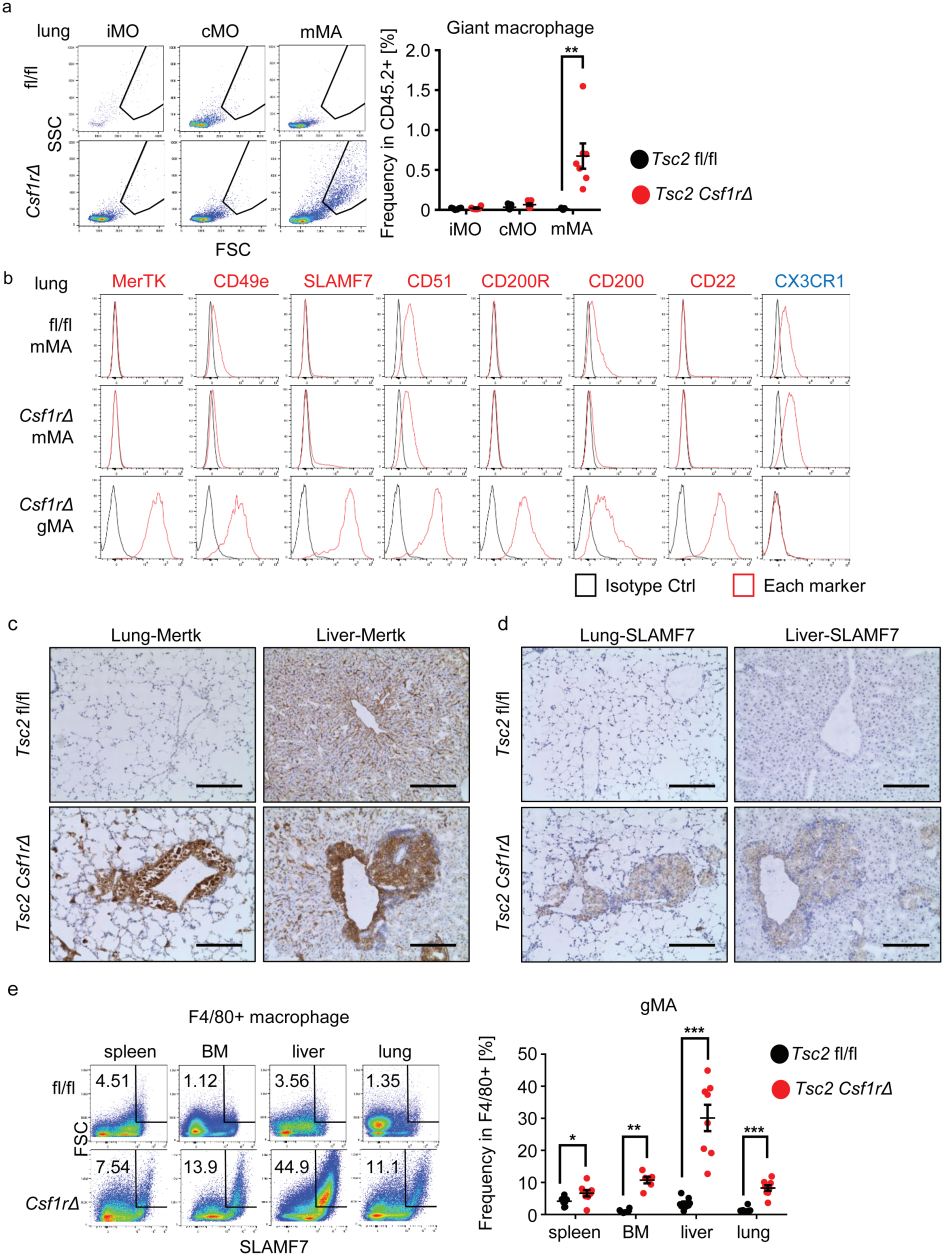
gMAs accumulated in the lung and liver are distinct from mMAs. (a) Dot plots show the forward and side scatter of lung macrophages from the indicated mice. Forward and side scatter of iMOs, cMOs, and mMAs with gates for giant macrophages (gMAs). The percentage of gMAs in CD45-positive hematopoietic cells is also shown (*n* = 7). (b) Each histograms show expression of cell surface markers in lung mMAs and gMAs from the indicated mice. The staining with isotype control antibody is also presented. (c, d) Immunostaining of the lung and liver sections from the indicated mice to detect MerTK and Slamf7. (e) Dot plots showing forward scatter and Slamf7 expression in F4/80^+^ macrophages from the indicated organs of the indicated mice. The gates for gMAs and percentages of gMAs in F4/80^+^ macrophages from the indicated organs are also shown (*n* = 5–8). *P < .05, **P < .01 and ***P < .001. Scale bars, 200 µm.

The iMOs that migrated into the liver and lungs were likely to transform into granuloma macrophages. Therefore, we focused on the population of macrophages with high levels of forward scatter, which were found in the mMA subset in the lungs of *Tsc2*^csf1rΔ^ mice (Fig. 4a). We refer to these cells as gMAs. Because gMAs were similar to mMAs in Ly6C^low^ FcγRIV^+^ phenotypes, we studied the relationship between mMAs and gMAs. With antibody array analyses of hepatic gMAs and mMAs (Supplementary Fig. S4), we found that hepatic gMAs more highly expressed the phagocytic receptor MerTK, adhesion molecules (CD49e, SLAMF7, CD51), and the inhibitory receptors with the ligand (CD200R, CD200, CD22) (Supplementary Fig. S5). These markers were also highly expressed in lung gMAs in *Tsc2*^*csf1rΔ*^ mice but not in lung mMAs in *Tsc2*^*csf1rΔ*^ and wild-type mice (Fig. 4b). In contrast to these upregulated cell surface markers, the expression of the fractalkine receptor CX3CR1 decreased (Fig. 4b, Supplementary Fig. S5). Analyses of lung and liver sections showed that MerTK and SLAMF7 were also expressed in granulomas in *Tsc2*^csf1rΔ^ mice (Fig. 4c and d). FACS analyses showed that F4/80^+^ SLAMF7^+^ gMAs were observed predominantly in the liver (Fig. 4e), followed by the lungs and BM of the *Tsc2*^*csf1rΔ*^ mice. These results demonstrate that gMAs were distinct from mMAs in terms of the expression of cell surface markers and tissue distribution.

### gMAs are similar to granuloma macrophages in gene expression

**Figure 5.**
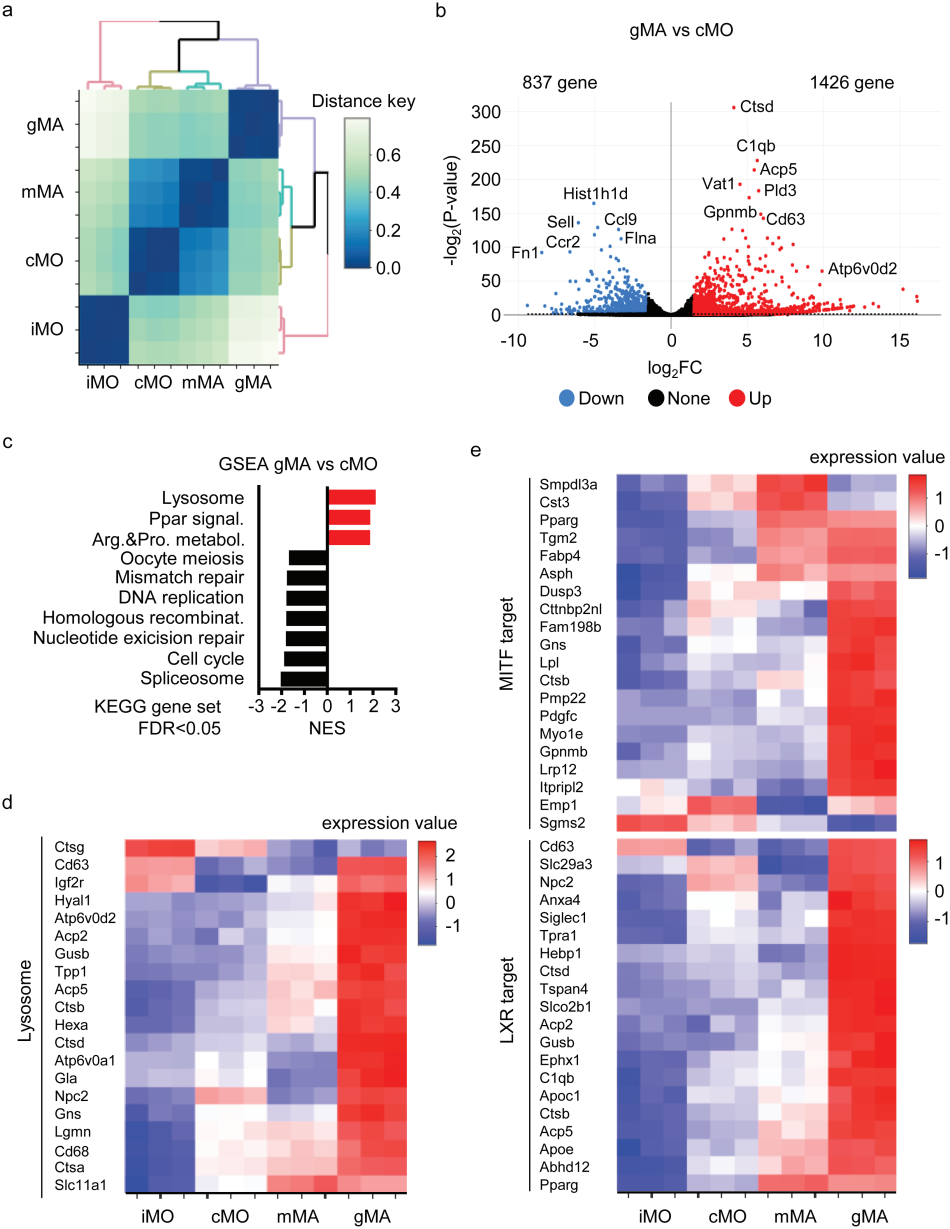
gMAs express target genes of MITF and LXRα. (a) Dendrogram showing hierarchical clustering of splenic iMOs, splenic cMOs, hepatic mMAs, and hepatic gMAs represented as distance key obtained from (1—correlation coefficient) based on transcriptome analyses. (b) Volcano plot showing the genes upregulated and downregulated by 2^1.5^ fold in comparison of hepatic gMAs to splenic cMOs. (c) GSEA of differentially expressed genes (DEGs) in hepatic gMAs compared with splenic cMOs. KEGG gene sets, which were positively and negatively enriched in hepatic gMAs with FDR lower than 0.05, are shown. (d, e) Heat maps showing the mRNA expression of the indicated genes in the indicated monocyte/macrophage subsets.

To compare splenic iMOs, splenic cMOs, hepatic mMAs, and hepatic gMAs in *Tsc2*^csf1rΔ^ mice, transcriptome analyses were performed. In terms of gene expression, hepatic gMAs were closer to hepatic mMAs and splenic cMOs than to splenic iMOs (Fig. 5a). Hepatic gMAs expressed 1728 and 1072 genes by 2^1.5^ fold higher or lower, respectively, than hepatic mMAs (Supplementary Fig. S6a). In the comparison between hepatic gMAs and splenic cMOs, hepatic gMAs expressed 1426 and 837 genes 2^1.5^ fold higher or lower, respectively (Fig. 5b). The “lysosome” gene set was positively enriched in DEGs when hepatic gMAs were compared to hepatic mMAs and splenic cMOs (Fig. 5c and Supplementary Fig. S6b). Many lysosome signature genes were more highly expressed in hepatic gMAs than in hepatic mMAs, splenic cMOs, and splenic iMOs (Fig. 5d). Highly expressed lysosomal genes included those involved in granuloma development. For example, overexpression of the lysosomal V-ATPase component Atp6v0d2 induces the transformation of macrophages into multinucleated macrophages (20) (Fig. 5b and d, Supplementary Fig. S6a). Granuloma macrophages in cardiac sarcoidosis also highly express lysosome-associated genes (10, 21), suggesting that gMAs are similar to the granuloma macrophages in cardiac sarcoidosis.

Next, we analyzed the gene expression profiles using the search engine “Enrichr” (10, 22, 23). The analyses based on the “All RNA-seq and ChIP-seq sample and signature search 4 (ARCHS4)” database suggested activation of transcription factors such as Nrf2, Irf8, Smrt, Ncor, Lxr, Mitf, and Vdr in hepatic gMAs when compared to hepatic mMAs (Supplementary Fig. S6c). We were interested in MITF and LXR because these transcription factors are also activated in granuloma macrophages in cardiac sarcoidosis (10, 21). Hepatic gMAs more highly expressed the target genes of MITF and LXRα than hepatic mMAs, splenic cMOs, and splenic iMOs (Fig. 5e). For example, MITF target genes included *Gpnmb* and *Gns*, whereas LXRα target genes included *Nr1h3* and *Apoe* (Supplementary Fig. S6d). Notably, GPNMB is a marker of multinucleated giant cells (11). These results further demonstrate the similarity between gMAs in *Tsc2*^*Csf1rΔ*^ mice and granuloma macrophages in sarcoidosis.

### gMA-like macrophages in sarcoidosis

**Figure 6.**
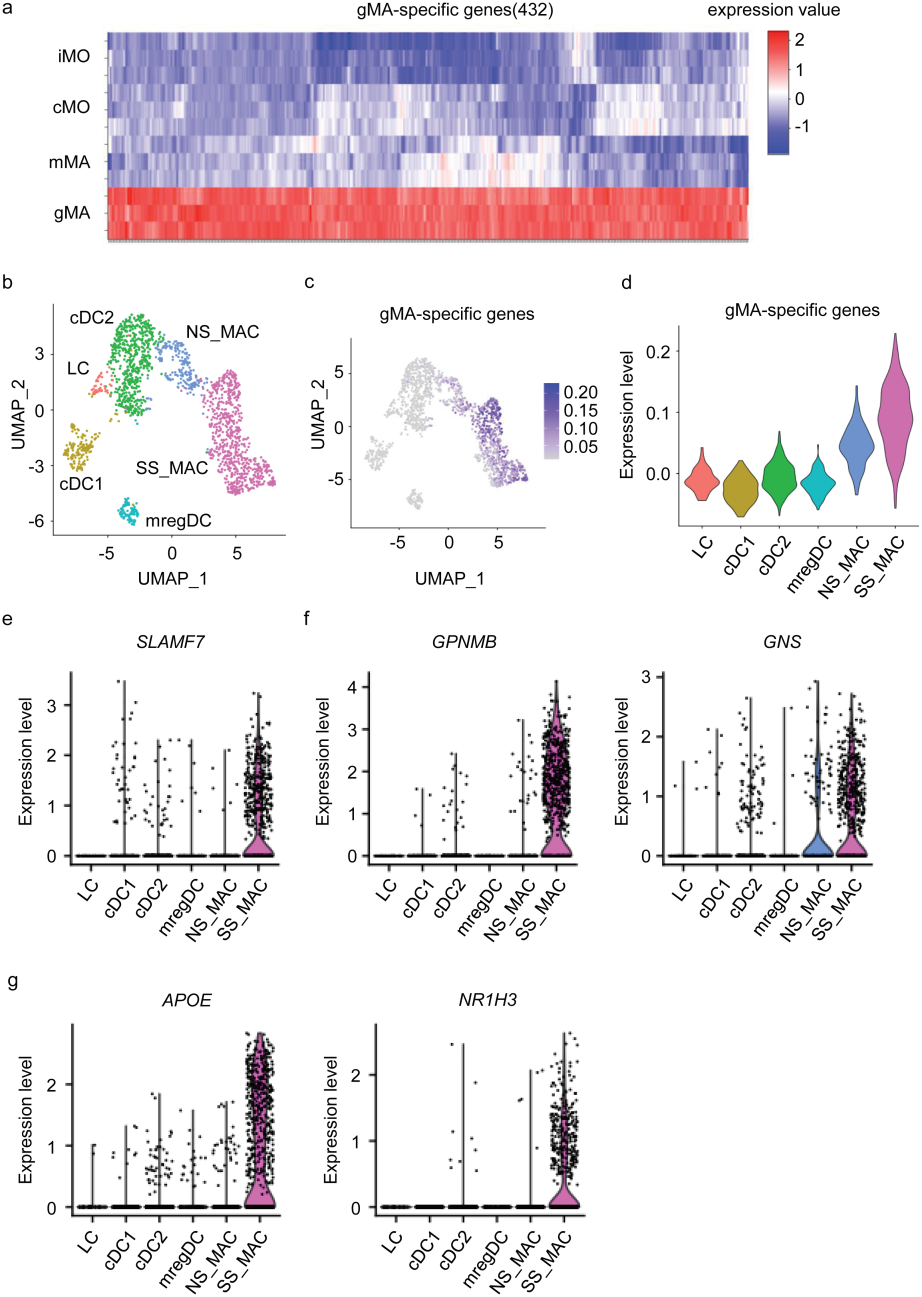
Sarcoidosis macrophages express gMA-specific genes. (a) Heat map showing the expression of the gMA-specific 432 genes in iMOs, cMOs, mMAs, and gMAs. (b) UMAP plots showing DC and macrophage clusters from three sarcoidosis patients and five healthy subjects. LC, Langerhans cells, cDC1 conventional type 1 dendritic cells; cDC2, conventional type 2 dendritic cells; mregDC, mature dendritic cells enriched in immunoregulatory molecules; NS_MAC, normal skin resident macrophages; SS_MAC, sarcoidosis skin resident macrophages. (c) UMAP plot showing the average expression levels of gMA-specific genes in DCs and macrophages. (d–g) Violin plots show the mean (d) and individual (e–g) expression of indicated genes in myeloid cell subsets.

To determine whether gMA-like macrophages accumulate in the granulomas of sarcoidosis patients, we focused on the gMA-specific 432 genes that were selected using the following two criteria (Fig. 6a, Supplementary Fig. S7). First, we focused on 2025 genes that were more highly expressed in hepatic gMAs with FDR less than 0.1 compared to splenic iMOs, splenic cMOs, and hepatic mMAs. The 2025 genes were narrowed down to 432 genes that were expressed in gMAs by 2^1.5^ fold higher than in splenic iMOs, splenic cMOs, and hepatic mMAs. The expression of hepatic gMA-specific genes in sarcoidosis granulomas was studied using the scRNA-seq results analyzing skin lesions from patients with sarcoidosis (GSE234901) (24). The gMA-specific genes were more highly expressed in sarcoidosis skin-resident macrophages than in normal skin-resident macrophages (Fig. 6b–d). The highly expressed genes included *SLAMF7* and the target genes of MITF and LXR, such as *GPNMB*, *GNS*, *APOE*, and *NR1H3* (LXRα) (Fig. 6e–g). These results suggest that hepatic gMAs in *Tsc2*^*Csf1rΔ*^ mice are similar to the granuloma macrophages in sarcoidosis.

## Discussion

In *Tsc2*^*Csf1rΔ*^ mice, Tsc2-deficiency increased the percentage of MPs such as CMPs, GMPs, cMoPs/MPs, and iMoPs, and of CD11b^+^ Ly6C^int^ CX3CR1^low^ CD14^−^ iMOs in the spleen. In wild-type mice, MPs and iMOs reside in the BM and must mature up to Ly6C^hi^ CX3CR1^hi^ cMOs before egressing from the BM. In *Tsc2*^*Csf1rΔ*^ mice, MPs and iMOs egressed from the BM and migrated to the spleen. iMOs in the BM and spleen of *Tsc2*^*Csf1rΔ*^ mice showed a lower expression of the maturation marker CD14 than BM iMOs in wild-type mice, suggesting that iMOs in *Tsc2*^*Csf1rΔ*^ mice were more immature than those in the BM of wild-type mice. The adoptive transfer of splenic iMOs from *Tsc2*^*Csf1rΔ*^ mice into wild-type mice resulted in granulomas in the liver and lungs, demonstrating that iMOs migrated into the liver and lungs to form granulomas. These results indicate that altered monocytopoiesis generates and delivers granuloma-forming iMOs to target organs, such as the liver and lungs.

Emergency hematopoiesis, a state characterized by myeloid-biased cell production and extramedullary hematopoiesis (25–28), is observed during infection. *Escherichia coli* infection increases the number of CD11b^+^ CXCR4^+^ premonocytes not only in the BM but also in circulation to replenish the peripheral pool of macrophages (29). TLR ligands and inflammatory cytokines drive emergency hematopoiesis (28, 30). TLR7 activates the PI3K–mTORC1 axis to enhance myelopoiesis during infection (31). Our results demonstrated the proliferation of MPs and iMOs in the spleen of the sarcoidosis mouse model, suggesting that emergency hematopoiesis generates iMOs that give rise to granuloma. In addition to myelopoiesis, mTORC1 mediates granuloma development during mycobacterial infection, because TLR2-dependent cMoP maturation into multinuclear giant cells is inhibited by rapamycin (4). Sarcoidosis may share mTORC1-dependent granuloma development with mycobacterial infection. However, the mechanisms that activate mTORC1 might vary with disease. Mycobacterial infection activates the TLR2–mTORC1 axis (32), which drives granuloma development, whereas TSC1 mRNA levels are significantly decreased in patients with the progressive form of sarcoidosis (6).

In *Tsc2*^*Csf1rΔ*^ mice, MPs, such as CMPs, GMPs, cMoPs, and iMoPs moved to and accumulated in the spleen. Although CSF1R is not expressed in CMPs and GMPs, its transcription is likely to be activated because Tsc2 deletion in CMPs and GMPs was detected by FACS analysis. Consequently, mTORC1 activation in CMPs, GMPs, cMoPs/MPs and iMoPs drives proliferation and maturation in *Tsc2*^*Csf1rΔ*^ mice. Consistent with this, iMOs, as well as other descendants, such as neutrophils and neutrophil-like monocytes, accumulated in the spleen. In contrast, MDPs, CDPs, and their descendants did not accumulate in the spleen. These results suggest that mTORC1 activation promotes proliferation of neutrophils/MPs, but not DCs/MPs.

Although EdU^+^ proliferating MPs were detected in the spleen, their percentages were much less than those of proliferating iMOs in the spleen, suggesting that MPs rapidly mature into iMOs. Tsc2-deletion by the *Lyz2* Cre-driver results in granuloma formation in the lung, liver, and skin (6). Granulomas developed in *Tsc2*^*Csf1rΔ*^ mice much earlier than in *Tsc2*^*Lyz2Δ*^ mice. Monocyte progenitors poorly expressed *Lyz2* compared with premonocytes (29), which corresponded to iMOs in the present study. Tsc2-deletion in MPs may cause earlier granuloma development in Tsc2^Csf1rΔ^ mice than in *Tsc2*^*Lyz2Δ*^ mice.

mTORC1 has been reported to control myeloid differentiation (33). Tsc2-deletion by an inducible Mx-Cre-driver (*Tsc2*^MxΔ^) increased the percentage of Ly6C^hi^ CX3CR1^low^ monocytes, which seems to correspond to the iMOs in our study. Among progenitors, Lin^−^ Sca-1^+^ c-kit^+^ (LSK) cells and GMPs were increased, but progenitors at more mature stages, such as MDPs and cMoPs/MPs, did not increase. Lin^−^ c-kit^+^ progenitors, GMPs, MDPs, and cMoPs/MPs were observed in the spleen of *Tsc2*^MxΔ^ mice. These results are not exactly similar to the phenotypes observed in *Tsc2*^*Csf1rΔ*^ mice, probably because of the differences in Cre drivers. It would be interesting to study granuloma development in *Tsc2*^MxΔ^ mice and compare these mice with *Tsc2*^*Csf1rΔ*^ and *Tsc2*^*Lyz2Δ*^ mice.

Our results suggest that iMOs mature into cMOs during and after migrating to the lungs and liver. gMAs also accumulated in the liver, lung, and BM. gMAs were similar to granuloma macrophages in expression of cell surface markers such as SLAMF7 and MerTK. Taken together, iMOs are likely to mature into cMOs and then into gMAs to form granulomas in the liver and lungs. As gMAs also accumulated in the BM, granulomas may also develop in the BM of *Tsc2*^*Csf1rΔ*^ mice. Along this line, it is of note that the BM is one of the organs where granulomas develop in sarcoidosis (34). A larger number of gMAs accumulated in the liver than the lung and BM. Because tissue-specific signals control the functional status of tissue-resident macrophages (35), liver-specific signals may drive cMO maturation into gMAs in the liver. Endogenous LXR ligands such as oxysterols induce and maintain Kupffer cells in the liver (36). LXR was also suggested to be activated in gMAs because of upregulated expression of the LXR target genes in gMAs. IL-4 activates macrophage mTORC1, which induces oxysterol production (37). Oxysterol-activated LXR drives macrophage differentiation into M2 type macrophages. Considering that IL-4 is a well-studied granuloma-driving signal (38), mTORC1-dependent production of oxysterols might drive granuloma formation in the livers of *Tsc2*^*Csf1rΔ*^ mice.

Transfer of iMOs, but not cMOs, from *Tsc2*^*Csf1rΔ*^ mice gave rise to granulomas in the lungs and livers of wild-type mice. Because ~3% of splenic iMOs proliferated, some of the transferred splenic iMOs were likely to proliferate in the spleen before migrating into the liver and lungs to give rise to granuloma. In contrast, less than 1% of splenic cMOs were in the S-phase. Transferred splenic cMOs may fail to give rise to granulomas because of poor proliferation. Alternatively, iMOs in the liver and spleen of *Tsc2*^*Csf1rΔ*^ mice matured into cMOs, suggesting that iMOs migrated into the liver and spleen before maturing into cMOs. cMOs in *Tsc2*^*Csf1rΔ*^ mice may not migrate into the liver and lungs as much as iMOs.

Transcriptome analyses demonstrated that mRNA expression of MITF target genes was upregulated in gMAs compared to cMOs and mMAs. MITF drives lysosomal biogenesis (39), which may explain the positive enrichment of lysosome-associated genes in gMAs compared to cMOs. Since MITF is negatively regulated by mTORC1 (39), mTORC1 activation and upregulation of lysosomal function in gMAs seem inconsistent. However, Tsc1-depetion in keratinocytes upregulates lysosomal biogenesis by activating the microphthalmia family of transcription factors, such as MITF (40). mTORC1 activates these transcription factors by inhibiting AKT, which negatively regulates the microphthalmia family of transcription factors. Activated MITF likely increased its target gene GPNMB, which is a marker of multinucleated giant cells (10). GPNMB in osteoclasts is involved in giant cell transformation and cell fusion (41). MITF also upregulates the expression of Atp6v0d2, a component of the V-ATPase (42). Overexpression of Atp6v0d2 in macrophages induces their transformation into multinucleated macrophages (20). These MITF target genes in gMAs may drive granuloma development in *Tsc2*^*Csf1rΔ*^ mice.

As gMA-like macrophages were detected in the skin lesions of sarcoidosis, monocyte/macrophage maturation into gMAs would occur also in sarcoidosis. Granuloma development might be inhibited by gMA depletion. Using an antibody array, we found a number of cell surface markers specifically expressed on gMAs but not on mMAs. These markers may serve as targets for gMA depletion in sarcoidosis. The antibody targeting SLAMF7, Elotuzumab, has been approved for the treatment of patients with previously treated multiple myeloma (43). This antibody may be useful for the treatment of refractory or multidrug-resistant sarcoidosis.

## Supplementary Material

dxad054_suppl_Supplementary_Figures_S1-S7
