# Supplementary material for: Aberrant monocytopoiesis drives granuloma development in sarcoidosis
Source: Int Immunol. 2023 Dec 26;36(4):183–96. doi: 10.1093/intimm/dxad054 (PMC10935646; doi:10.1093/intimm/dxad054)
Supplement: dxad054_suppl_Supplementary_Figures_S1-S7 [file dxad054_suppl_supplementary_figures_s1-s7.docx]

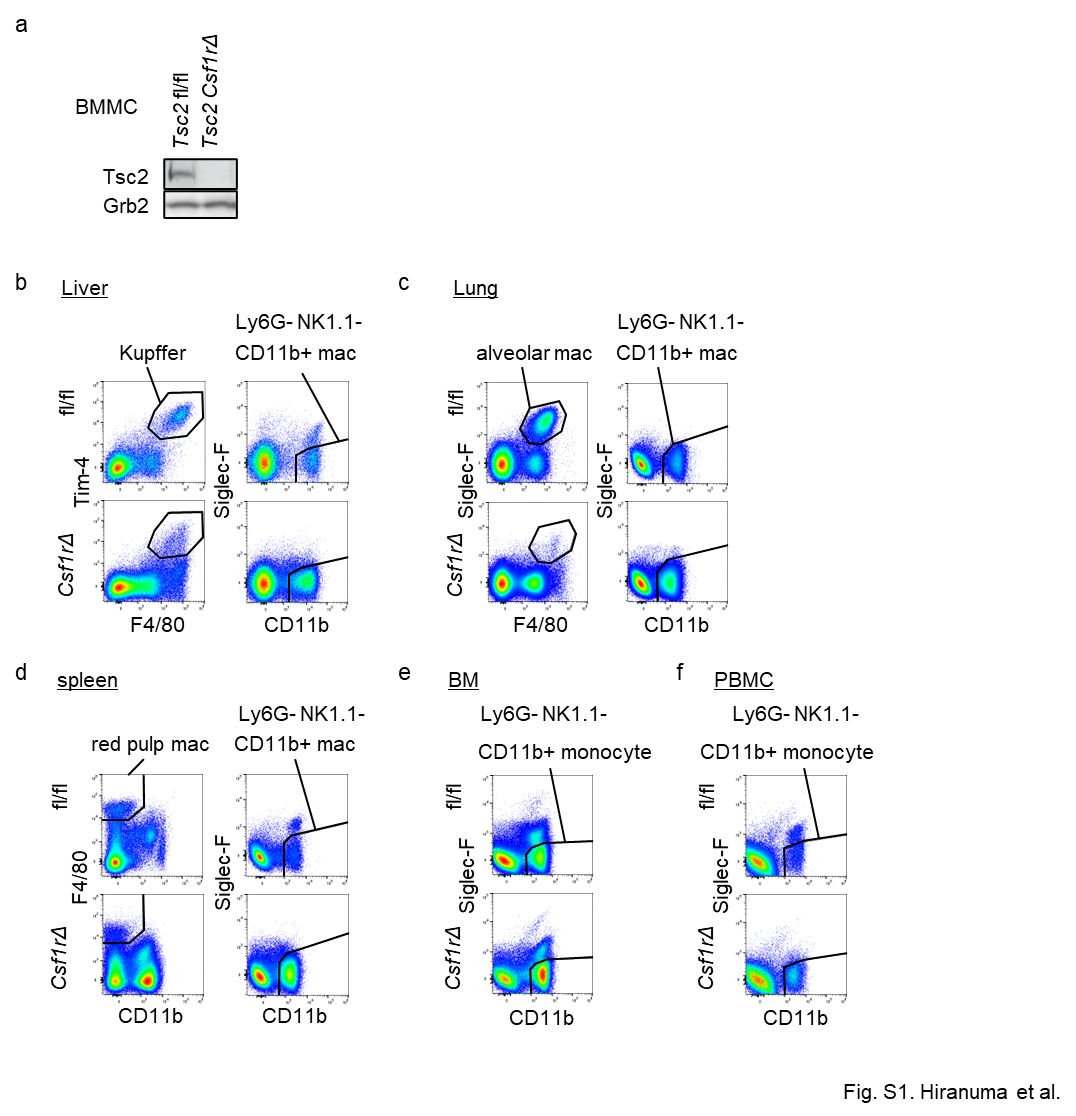


**Figure S1. TSC2 deletion in BM-macrophages from *Tsc2^Csf1rΔ^* mice**

**a**, Immunostaining of TSC2 and Grb2 as loading controls in BM-derived macrophage lysates from the indicated mice. **b-f**, Dot plots showing the expression of Tim-4, F4/80, CD11b, and Siglec-F in CD45^+^cells from the liver (**b**), lungs (**c**), spleen (**d**), BM (**e**), and peripheral blood (**f**) from the indicated mice.

**
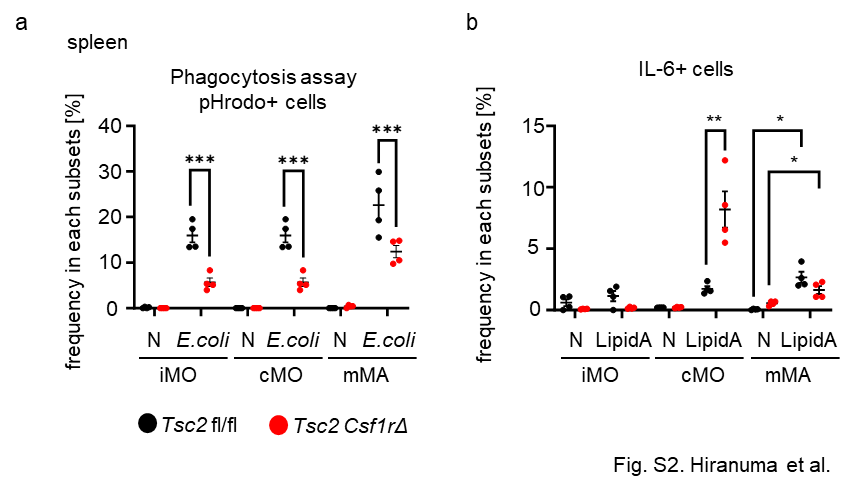
**

**Figure S2.** Phagocytic activity and cytokine production of monocyte/macrophages

**a**, Percentage of pHrodo Green+ iMOs, cMOs, and mMAs in splenic cells treated with 50 µg/mL pHrodo Green *E.coli* for 1h (n = 4). **b**, Percentage of IL-6+ iMOs, cMOs, and mMAs in splenic cells treated with Brefeldin A (N) or Brefeldin A with 1µg/mL Lipid A (Lipid A) for 3h (n = 4). Two-way ANOVA. *P<0.05, **P<0.01, ***P<0.001

**
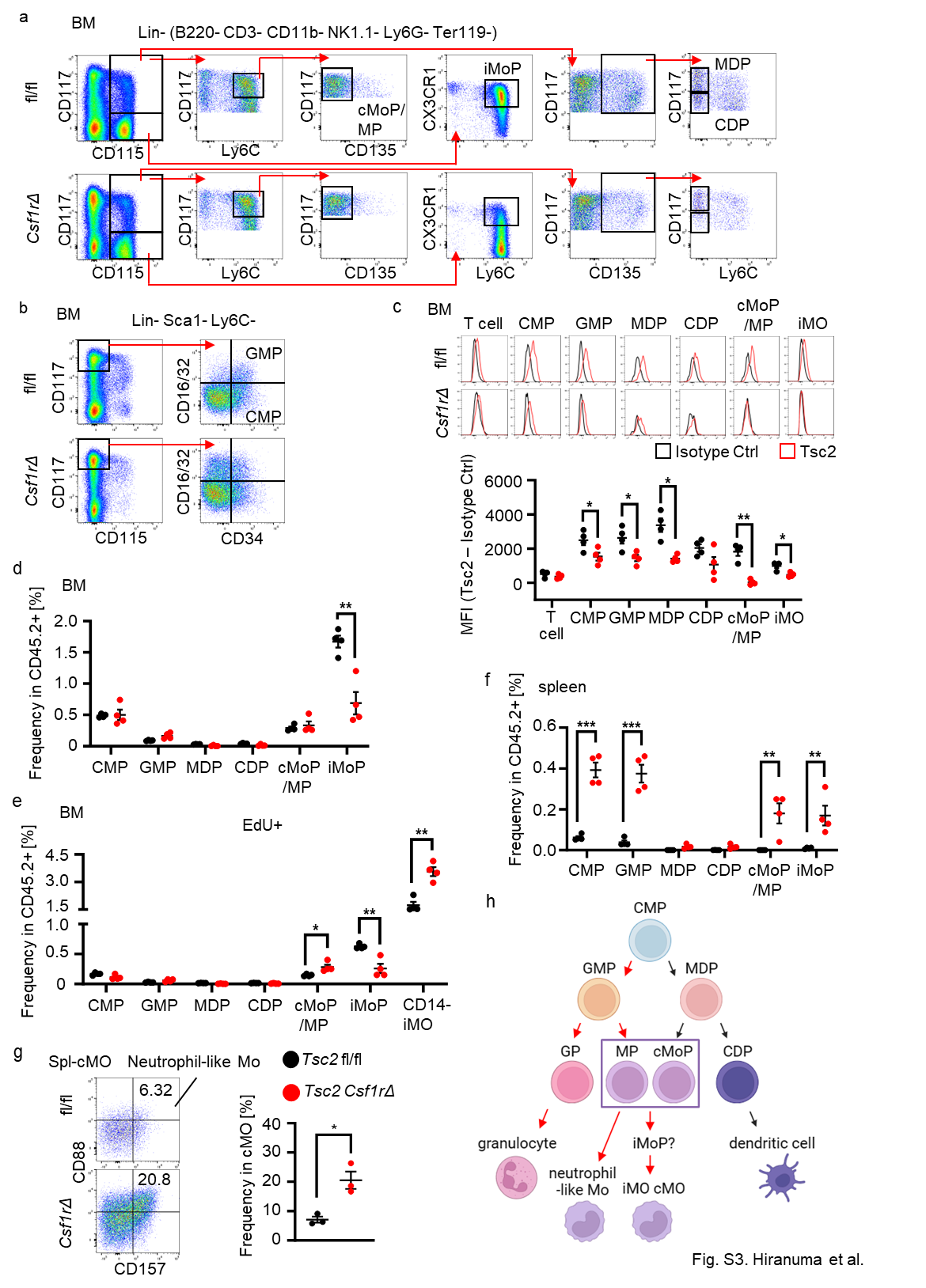
**

**Figure S3.** monocyte progenitors in the BM of *Tsc2^Csf1r^*^Δ^ mice.

**a**, Dot plots showing the expression of CD117, CD115, Ly6C, and CD135 in lineage-negative cells in the BM of the indicated mice. **b**, Dot plots show the expression of CD117, CD115, CD34, and CD16/32 in lineage-negative, Sca-1^-^, and Ly6C^-^ cells in the BM of the indicated mice. **c**, Gray and red histograms show representative staining of T cells, indicated myeloid progenitors and immature monocytes in the BM of indicated mice with isotype control Ab and anti-Tsc2 Ab, respectively. The mean fluorescence intensity (MFI) of the staining against Tsc2 was subtracted by the MFI of Isotype Ctrl staining. Dot plots show subtracted MFI (n = 4). T cells in BM are shown as a control. **d**, Percentage of indicated monocyte progenitors in the BM of the indicated mice (n = 4). **e**, Percentages of EdU+ CMPs, GMPs, MDPs, CDPs, cMoPs/MPs, iMoPs and CD14- iMOs in the BM of indicated mice (n = 4). **f**, Percentages of indicated monocyte progenitors and monocytes in the spleen from the indicated mice (n = 4). **g**, Dot plots show the expression of CD157 and CD88 on cMOs in the spleen of the indicated mice. The percentage of neutrophil-like monocytes is shown (n = 4). *P<0.05, **P<0.01, ***P<0.001 **h**, Differentiation of progenitors for neutrophils/monocytes and dendritic cells is shown. Differentiation pathways upregulated in in *Tsc2^Csf1rΔ^* mice are shown by red arrows.

**
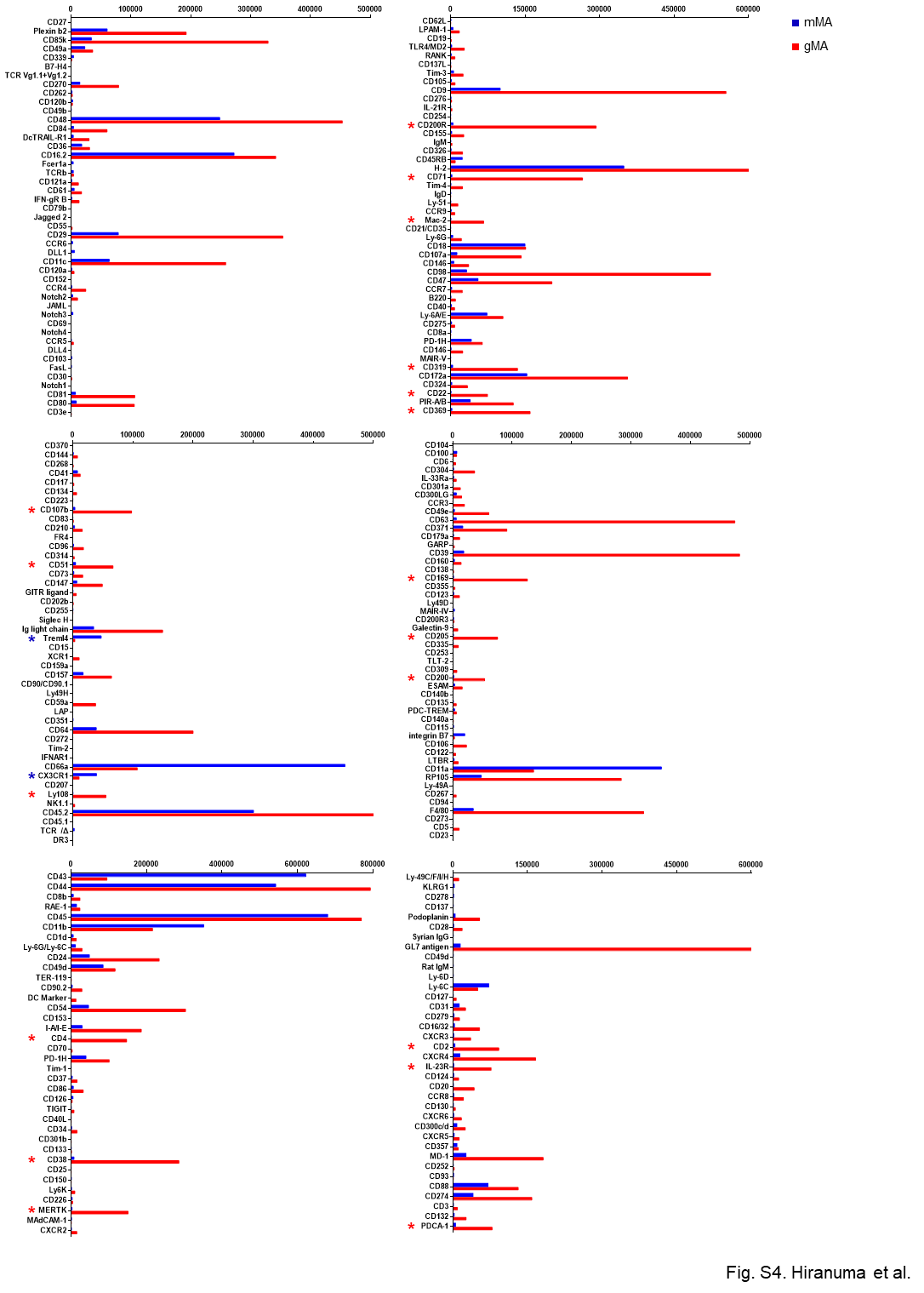
Figure S4 antibody array analyses of mMAs and gMAs from the liver of *Tsc2^Csf1rΔ^* mice**

Mean fluorescence intensity of staining with antibodies against the indicated antigens. Blue and Red bars show staining of mMAs and gMAs, respectively, in the livers of *Tsc2^Csf1rΔ^* mice.

**
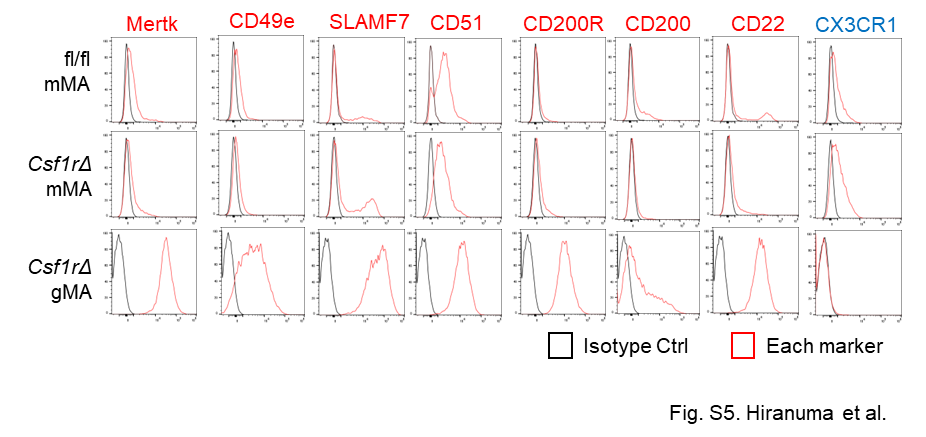
**

**Figure S5 Differential expression of cell surface markers on mMAs and gMAs**

Red and black Histograms show staining of indicated macrophages from indicated mice with indicated and isotype control Abs.

**
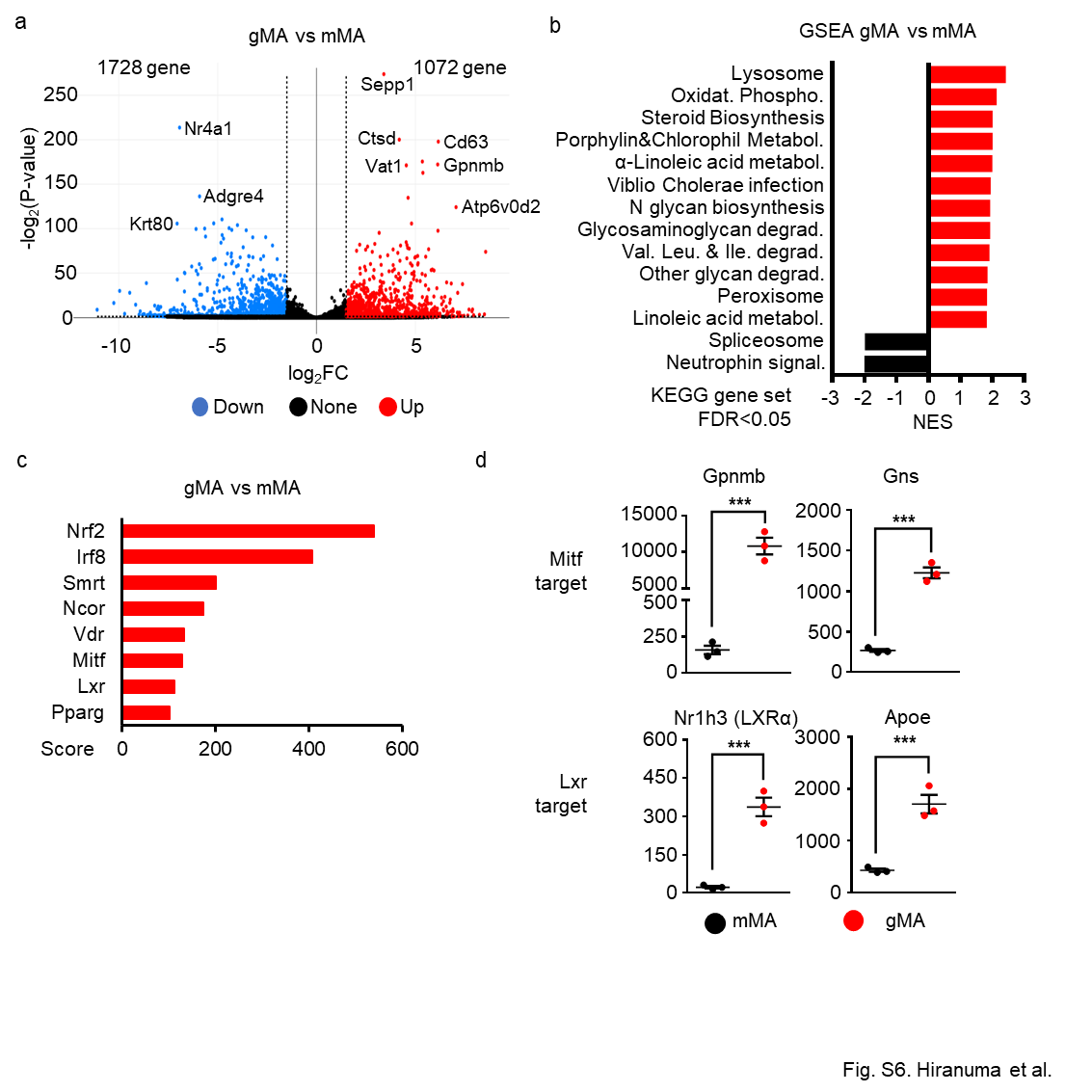
**

**Figure S6. Differential gene expression in gMAs and mMAs**

**a**, Volcano plots show genes expressed in hepatic gMAs from *Tsc2^Csf1rΔ^* mice 2^1.5^ fold higher (red) or lower (blue) than in hepatic mMAs. **b**, GSEA comparing hepatic gMAs vs. hepatic mMAs. Bars indicate gene sets that were positively (red) or negatively (black) enriched in hepatic gMAs. **c**, The bars show the transcription factors that were predicted to be activated in hepatic gMAs when compared to hepatic mMAs. **d**, Dot plots show reads per million (RPM) values of the indicated genes in hepatic gMAs (red) and hepatic mMAs (black).

**
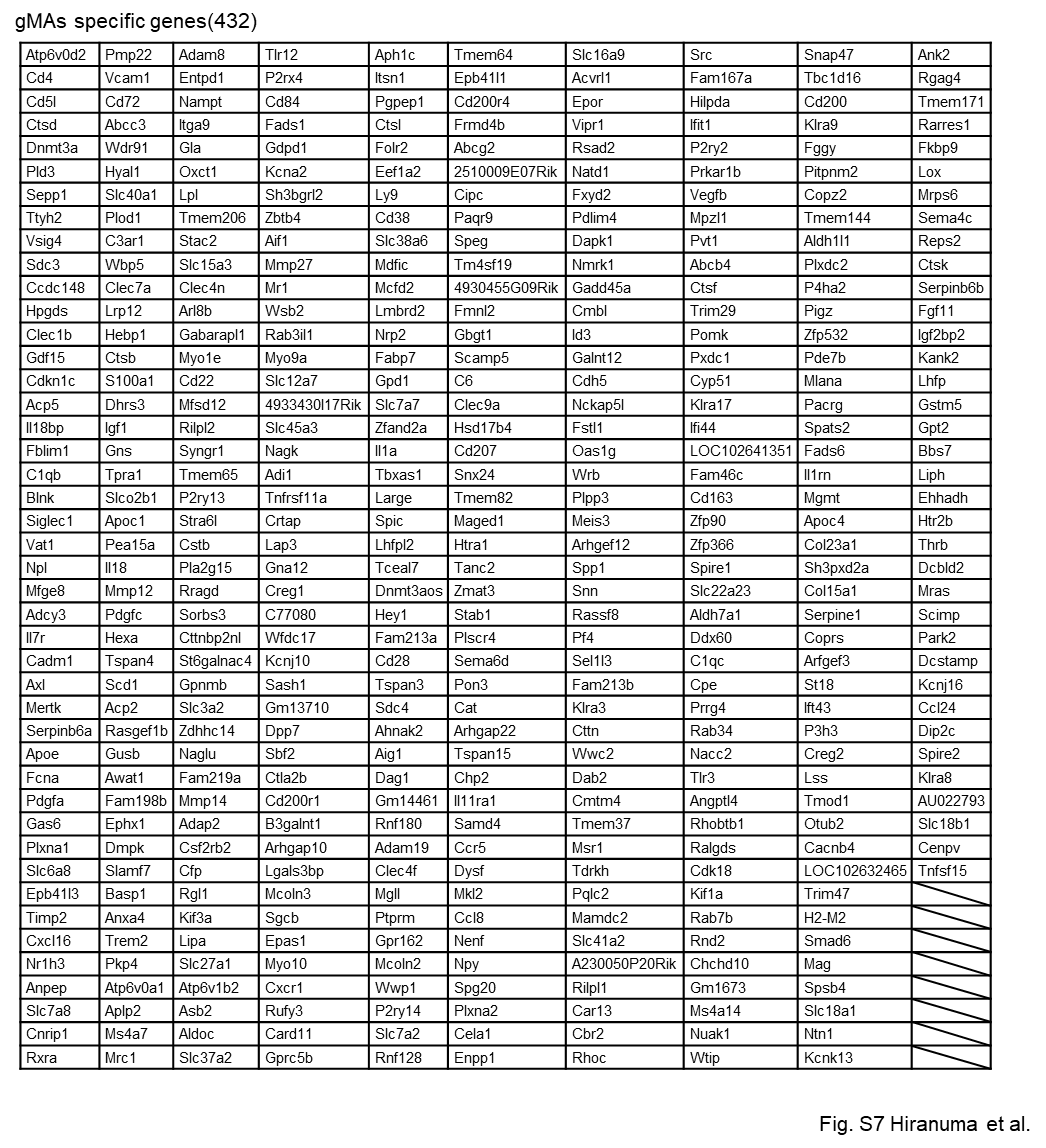
Figure S7 gMA-specific genes**

List of gMA-specific genes.
